# Genome‑wide profiling of DNA methylation and gene expression unravel the epigenetic landscape in diabetes-related hypothyroidism

**DOI:** 10.1186/s13148-021-01109-2

**Published:** 2021-06-06

**Authors:** Jingyi Luo, Xiaoxia Wang, Li Yuan, Lixin Guo

**Affiliations:** 1 Department of Endocrinology, The Key Laboratory of Geriatrics, Beijing Institute of Geriatrics, Beijing Hospital, National Center of Gerontology, National Health Commission, Institute of Geriatric Medicine, Chinese Academy of Medical Sciences, No.1 DaHua Road, Dong Dan, Beijing, 100730 People’s Republic of China; 2 The Savaid School of Medicine, University of Chinese Academy of Sciences, Beijing, People’s Republic of China

**Keywords:** Diabetes-related hypothyroidism, DNA methylation, Gene expression, Epigenetic modification

## Abstract

**Background:**

Type 2 diabetes mellitus (T2DM) and hypothyroidism are two common endocrine diseases and the phenomenon that the prevalence of diabetes-related hypothyroidism shows a significant upward trend deserves further attention, but the specific pathogenesis is not yet clear. The study aimed to explore the molecular mechanisms on DNA methylation regulating gene expression and participating in diabetes-related hypothyroidism through genome-wide DNA methylation and RNA sequencing.

**Results:**

The prevalence of hypothyroidism in T2DM patients was significantly higher than that in patients without T2DM (*P* = 0.018). Meanwhile, high TSH and low T3 and T4 levels were detected in diabetic mice. Low T3 and T4 levels were detected in Nthy-ori3-1 cells incubated in high-glucose medium. Differentially expressed genes (DEGs) and differentially methylated regions (DMRs) were detected by RNA sequencing and reduced representation bisulfite sequencing in Nthy-ori3-1 cells cultured in high-glucose and normal medium. Functional enrichment analyses reveled that DMRs and DEGs were related to significant pathways including Ras, Wnt and MAPK pathways.

**Conclusions:**

We observed the potential connection between T2DM and hypothyroidism. This study was the first one carrying out DNA methylation and gene expression profiles to explore epigenetic modification in diabetes-related hypothyroidism, which provided information for the detailed study of the molecular mechanism in diabetes-related hypothyroidism.

**Supplementary Information:**

The online version contains supplementary material available at 10.1186/s13148-021-01109-2.

## Background

The prevalence of diabetes mellitus is increasing worldwide. There were approximately 425 million adults living with diabetes mellitus worldwide in 2017 according to data from the International Diabetes Federation and the number is expected to reach 629 million by 2045 [1]. It is worth noting that diabetes mellitus has become a major public health problem in the general population of China. Approximately 11.6% of Chinese adults was estimated to have diabetes mellitus in 2010 [2]. Long-term hyperglycemia can lead to complications such as diabetic nephropathy, diabetic retinopathy, diabetic neuropathy, atherosclerosis, and adversely affect patients' health and quality of life [3–5].

Hypothyroidism is a common endocrine problem caused by the reduction of thyroid hormone synthesis and secretion or insufficient tissue utilization due to various reasons. A study of thyroid diseases in 10 cities of China completed in 2011 (*n* = 15,008) showed that the prevalence of hypothyroidism among people over 18 years old was 17.73% [6]. Hypothyroidism has been found be associated with atherosclerosis, myocardial infarction and heart failure, requiring life-long replacement therapy with thyroid hormones [7–9].

T2DM and hypothyroidism are two frequently occurring disorders involving the endocrine system and the relationship between them was first reported in 1979 [10]. Since then, many studies have observed the coexistence of diabetes mellitus and hypothyroidism. A study in Mexico compared 1848 patients with T2DM and 3313 non-diabetic patients, and the results showed that the odd ratio (OR) of the prevalence of hypothyroidism in T2DM patients was 3.45 (95% CI 2.5–4.7) [11]. A systematic review and meta-analysis reported by Han et al. compared 5768 T2DM patients with 6225 non-diabetic patients. It was found that subclinical hypothyroidism was more common in T2DM patients with an OR of 1.93 (95% CI 1.66–2.24) [12]. Meanwhile, hypothyroidism can lead to high blood pressure and hyperlipidemia, affect insulin secretion, damage microvascular and macrovascular function, thereby increasing the risk of diabetic complications and exacerbating the progression of diabetes [1]. Diabetic complications in patients with subclinical hypothyroidism were more common than them in people with normal thyroid function. The overall OR for diabetic nephropathy, diabetic retinopathy, peripheral arterial disease and diabetic peripheral neuropathy was 1.74 (95% CI 1.34–2.28), 1.42 (95% CI 1.21–1.67), 1.85 (95% CI 1.35–2.54) and 1.87 (95% CI 1.06–3.28), respectively [12]. Therefore, the specific molecular mechanism of diabetes-related hypothyroidism needs to be studied urgently.

DNA methylation an epigenetic modification pattern in which methyl groups are added to the cytosine residues at the context of CpG dinucleotides, thereby changing the transcription activity of DNA fragments without changing the DNA sequence [13]. Environmental factors could regulate gene expression and function through epigenetic factors [14]. Epigenetic modification has opened up a new research field in the study of diabetes mellitus and thyroid diseases, but we still know little about how DNA methylation participates in the pathogenesis of diabetes-related hypothyroidism through regulating gene expression. Therefore, genome‑wide profiles of DNA methylation and gene expression were detected in the current study and we attempted to clarify the underlying mechanism of diabetes-related hypothyroidism with regard to epigenetics.

## Results

### Prevalence of thyroid dysfunction in patients with T2DM

A total of 1551 patients with T2DM and 374 healthy controls were included in the study. The prevalence of overt hypothyroidism, subclinical hypothyroidism, overt hyperthyroidism and subclinical hyperthyroidism were 1.74%, 3.93%, 0.19% and 1.93%, respectively (Table 1). The results showed that the prevalence of hypothyroidism in T2DM patients was significantly higher than that in patients without T2DM (*P* = 0.018). In contrast, the prevalence of hyperthyroidism in T2DM patients was lower than that in healthy controls (*P* < 0.001).

**Table 1:** Prevalence of thyroid disorders in patients with and without type 2 diabetes mellitus

	Patients with T2DM *n* (%)	Controls *n* (%)	*P* value
Hypothyroidism	88 (5.67)	10 (2.67)	0.018
Subclinical hypothyroidism	61 (3.93)	9 (2.41)	0.157
Overt hypothyroidism	27 (1.74)	1 (0.27)	0.033
Hyperthyroidism	33 (2.13)	22 (5.88)	< 0.001
Subclinical hyperthyroidism	30 (1.93)	16 (4.28)	0.008
Overt hyperthyroidism	3 (0.19)	6 (1.60)	< 0.001

### Thyroid dysfunction in diabetic mice

**Fig. 1:**
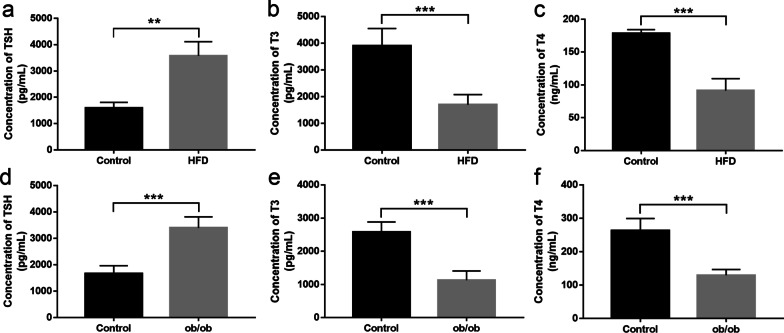
Thyroid function in diabetic mice. High TSH (**a**), low T3 (**b**) and T4 (**c**) levels were detected in the serum of HFD-fed diabetic mice (*n* = 5 per group). High TSH (**d**), low T3 (**e**) and T4 (**f**) levels were detected in the serum of ob/ob mice (*n* = 5 per group). Data are presented as mean ± SD. **P < 0.01, ***P < 0.001

Blood was collected from high-fat-diet (HFD) -fed mice and ob/ob mice after anesthesia was administered. Serum T3, T4 and TSH concentrations were detected by ELISA. Serum TSH level was significantly higher in HFD-fed mice (Fig. 1a, *P* = 0.001) and ob/ob mice (Fig. 1d, *P* < 0.001). At the same time, concentration of T3 was markedly lower in HFD-fed mice (Fig. 1b, *P* < 0.001) and ob/ob mice (Fig. 1e, *P* < 0.001). Serum T4 level was lower in HFD-fed mice (Fig. 1c, *P* < 0.001) and ob/ob mice (Fig. 1f, *P* < 0.001), either.

### Thyroid hormone secretion in Nthy-ori3-1 cells incubated in high-glucose medium

**Fig. 2:**
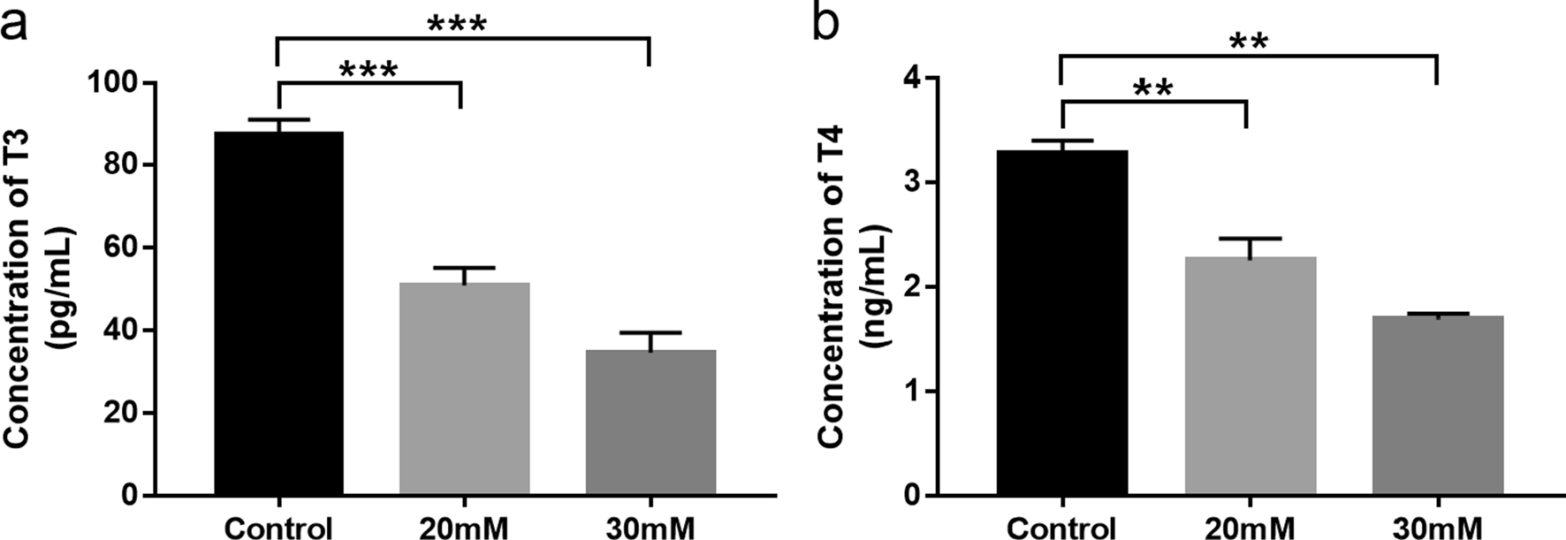
Thyroid hormone secretion in Nthy-ori3-1 cells incubated in high-glucose medium. High concentration of glucose reduced T3 (**a**) and T4 (**b**) levels in the culture supernatant of Nthy-ori3-1 cells. Data are presented as mean ± SD. **P < 0.01, ***P < 0.001

Nthy-ori3-1 cells were incubated in high-glucose medium and normal medium for 48 h. ELISA kits were used for detecting concentrations of T3 and T4 after the culture supernatant of Nthy-ori3-1 cells were collected. T3 (Fig. 2a, *P* < 0.001 when glucose concentration was 20 mM and 30 mM) and T4 (Fig. 2b, *P* = 0.004 when glucose concentration was 20 mM and *P* = 0.001 when glucose concentration was 30 mM) levels was lower in high-glucose groups compared to the control group.

### Differentially expressed genes (DEGs) in high-glucose-incubated Nthy-ori3-1 cells

**Fig. 3:**
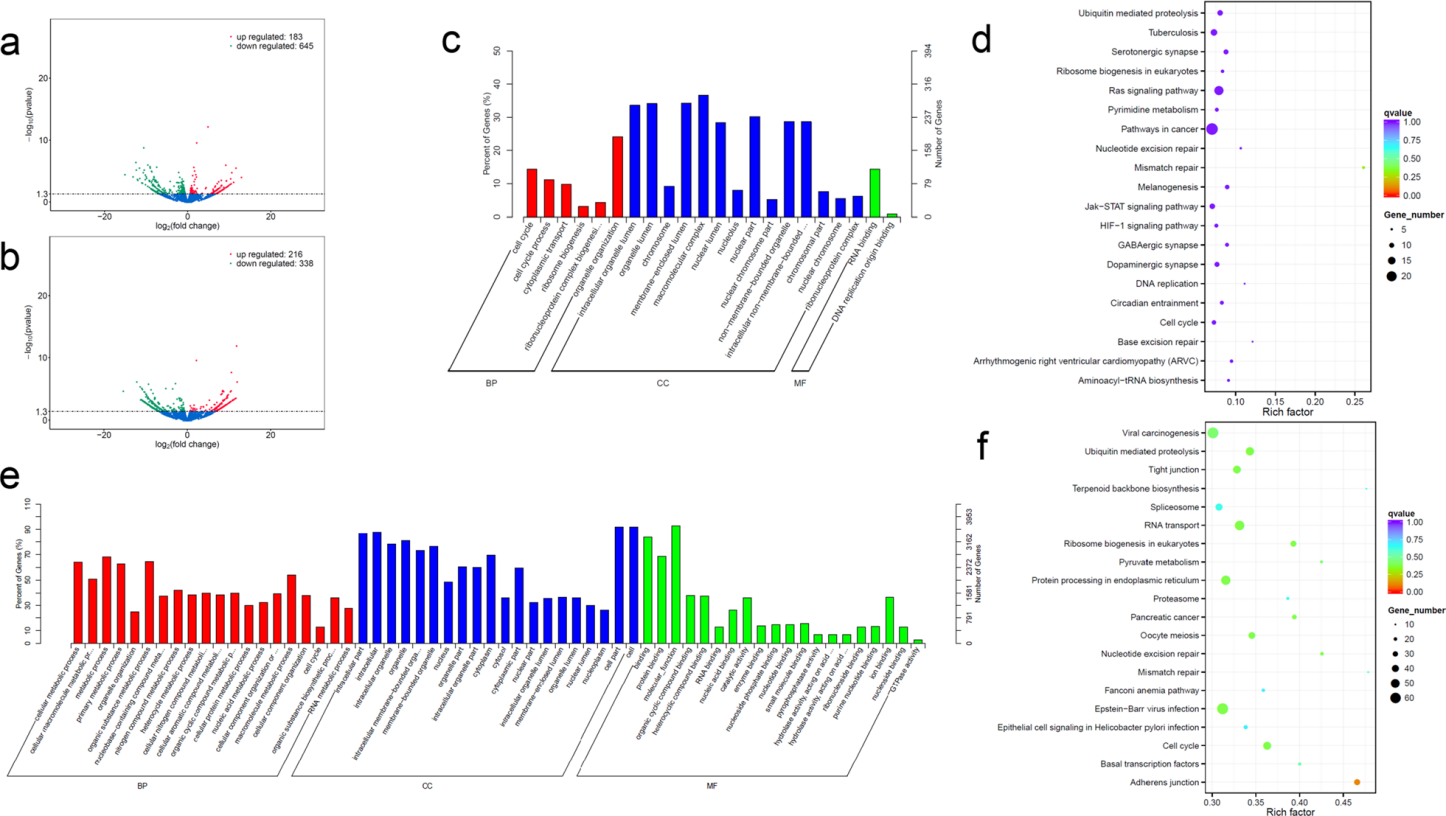
Functional enrichment of DEGs. Differentially expressed mRNAs and lncRNAs were shown by volcano plots (**a** and **b**). The red and green dots represented statistically upregulated and downregulated genes. Functional enrichment of mRNAs (**c** and **d**) and lncRNAs (**e** and **f**) were conducted using GO and KEGG pathway analyses, respectively

Changes in gene expression profile were found by next-generation sequencing between Nthy-ori3-1 cells incubated in high-glucose medium and normal medium. According to the coding ability of transcripts, they were divided into mRNAs and lncRNAs. The most dysregulated genes were shown in Table 2. Volcano plots of mRNA and lncRNA were drawn, respectively. A total of 183 mRNAs and 216 lncRNAs were significantly upregulated, and 645 mRNAs and 338 lncRNAs were significantly downregulated (Fig. 3a, b).

**Table 2:** The most upregulated and downregulated genes in Nthy-ori3-1 cells incubated in high-glucose medium

Gene ID	Gene name	Gene locus	Type	log2FoldChange	Regulation	*P* value
ENSG00000227359	AC017074.1	chr2:113677702-113704078	lncRNA	11.97	Up	8.75E−07
XLOC_089671	XLOC_089671	chr6:116076182-116100708	lncRNA	11.82	Up	1.38E−12
XLOC_000164	XLOC_000164	KI270442.1:318526-380002	lncRNA	11.69	Up	3.54E−04
XLOC_014988	XLOC_014988	chr10:91307296-91349366	lncRNA	11.46	Up	4.57E−04
XLOC_080045	XLOC_080045	chr5:48317804-48354889	lncRNA	11.14	Up	6.23E−04
XLOC_007405	XLOC_007405	chr1:41433266-41461157	lncRNA	− 11.03	Down	6.50E−04
XLOC_027534	XLOC_027534	chr13:75481300-75505587	lncRNA	− 11.22	Down	6.61E−04
ENSG00000250551	MIR583HG	chr5:96050115-96215519	lncRNA	− 11.31	Down	5.30E−06
XLOC_080208	XLOC_080208	chr5:58197873-58259549	lncRNA	− 12.12	Down	8.76E−07
ENSG00000240790	AC073934.1	chr7:127644685-128092784	lncRNA	− 15.36	Down	2.72E−05
ENSG00000249141	AL159163.1	chr6:166858094-166956124	mRNA	12.96	Up	1.11E−04
ENSG00000157654	PALM2-AKAP2	chr9:109780309-110172512	mRNA	11.64	Up	3.44E−06
ENSG00000266202	AC005697.1	chr17:27798806-27893365	mRNA	11.07	Up	6.71E−04
ENSG00000259171	AL163636.2	chr14:20684587-20700576	mRNA	10.63	Up	1.84E−05
ENSG00000112232	KHDRBS2	chr6:61679961-62286225	mRNA	10.48	Up	1.16E−03
ENSG00000285625	AC117378.1	chr12:56714612-56741535	mRNA	− 12.1	Down	2.27E−04
ENSG00000285245	AL162417.1	chr9:133098121-133163914	mRNA	− 12.36	Down	6.95E−06
ENSG00000198211	AC092143.1	chr16:89919165-89936092	mRNA	− 12.45	Down	4.16E−07
ENSG00000270757	HSPE1-MOB4	chr2:197500413-197550726	mRNA	− 13.14	Down	8.63E−05
ENSG00000284776	AL121900.2	chr20:18567453-18744216	mRNA	− 15.10	Down	3.96E−05

### Functional enrichment of DEGs

In order to better explore the function and molecular pathway of DEGs, functional enrichment was conducted using Gene Ontology (GO) and Kyoto Encyclopedia of Genes and Genomes (KEGG) analyses. Go analysis was carried out from three aspects: molecular function, biological process and cellular component. The significant GO clusters such as cell cycle, RNA binding, DNA replication origin binding for mRNAs (Fig. 3c) and cellular metabolic process, cellular protein metabolic process, protein binding for lncRNA-targeting genes (Fig. 3e) were found. Significant KEGG pathways related to ubiquitin-mediated proteolysis, jak-STAT signaling pathway and Ras signaling pathway were enriched for differentially expressed mRNAs (Fig. 3d). Enrichment pathways included RNA transport and cell cycle for lncRNA-targeting genes were also found (Fig. 3f).

### Differentially methylated regions (DMRs) in high-glucose-incubated Nthy-ori3-1 cells

**Fig. 4:**
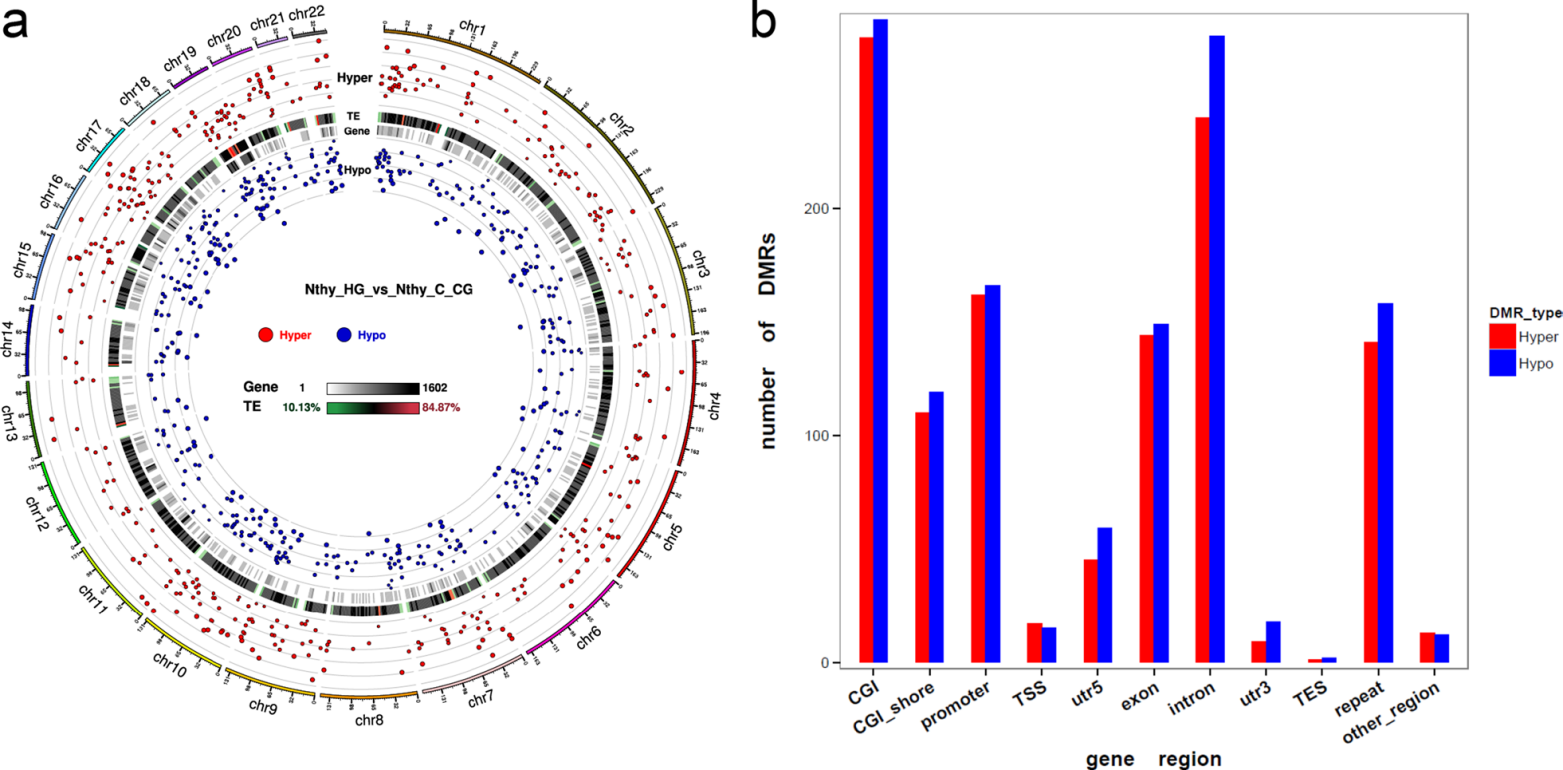
DMRs in high-glucose-incubated Nthy-ori3-1 cells. The significance and distribution of DMRs on the chromosome were displayed in a circular plot (**a**). The distribution of DMRs based on gene regions were shown in histogram (**b**). Hyper- and hypomethylated regions were colored in red and blue, respectively

A total of 2662 DMRs were identified, including 1272 hypermethylated and 1390 hypomethylated DMRs. The significance and distribution of DMRs on the chromosome were shown in Fig. 4a. Hyper- and hypomethylated DMRs were colored in red and blue, respectively. Distribution of DMRs was shown in Fig. 4b.

### Functional enrichment of DMRs -related genes

**Fig. 5:**
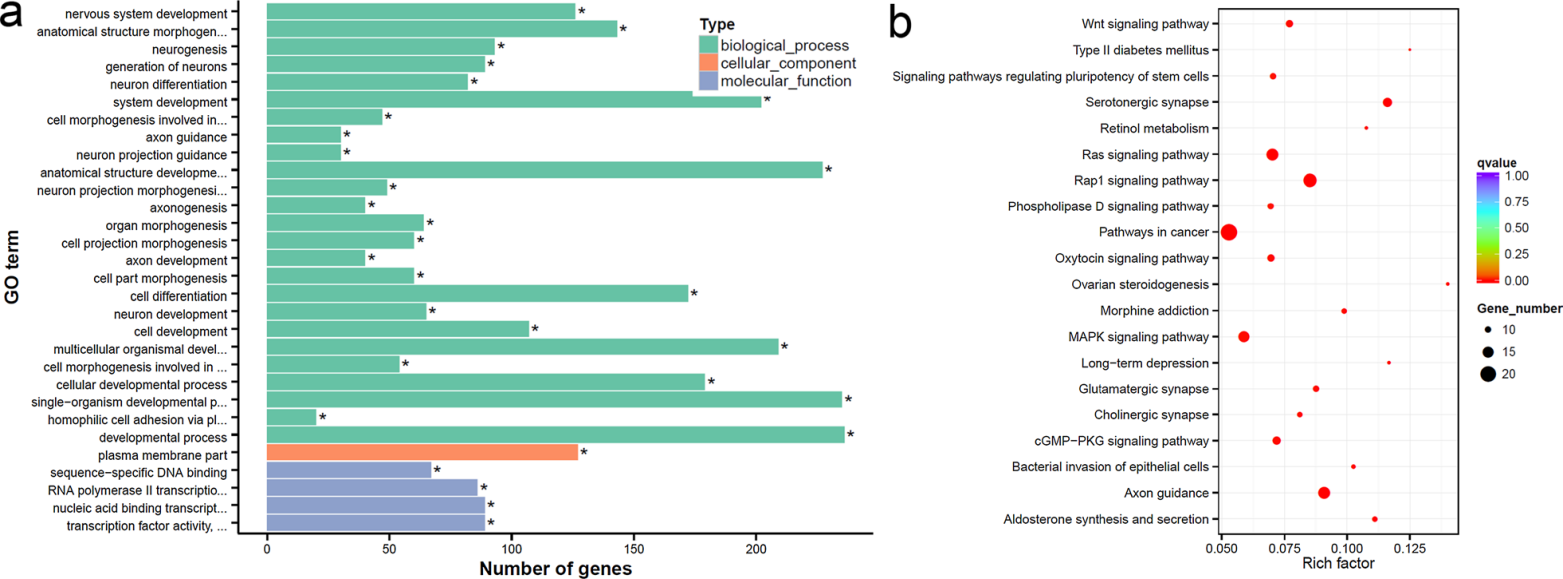
Functional enrichment of DMRs-related genes. GO (**a**) and KEGG (**b**) analyses of DMRs-related genes in Nthy-ori3-1 cells incubated in high-glucose medium versus controls

GO analysis for DMRs-related genes was conducted to explore biological processes and problems. The significant GO clusters such as cell development, cell differentiation and transcription process were enriched (Fig. 5a). Genes coordinated with each other to perform their biological functions. Through significant enrichment of pathways, the most important biochemical pathways and signal transduction pathways involved in DMR-related genes could be determined. Significant KEGG pathways related to Wnt, Ras and MAPK signaling pathways were enriched (Fig. 5b).

### Overlap analysis between DMRs and DEGs

**Fig. 6:**
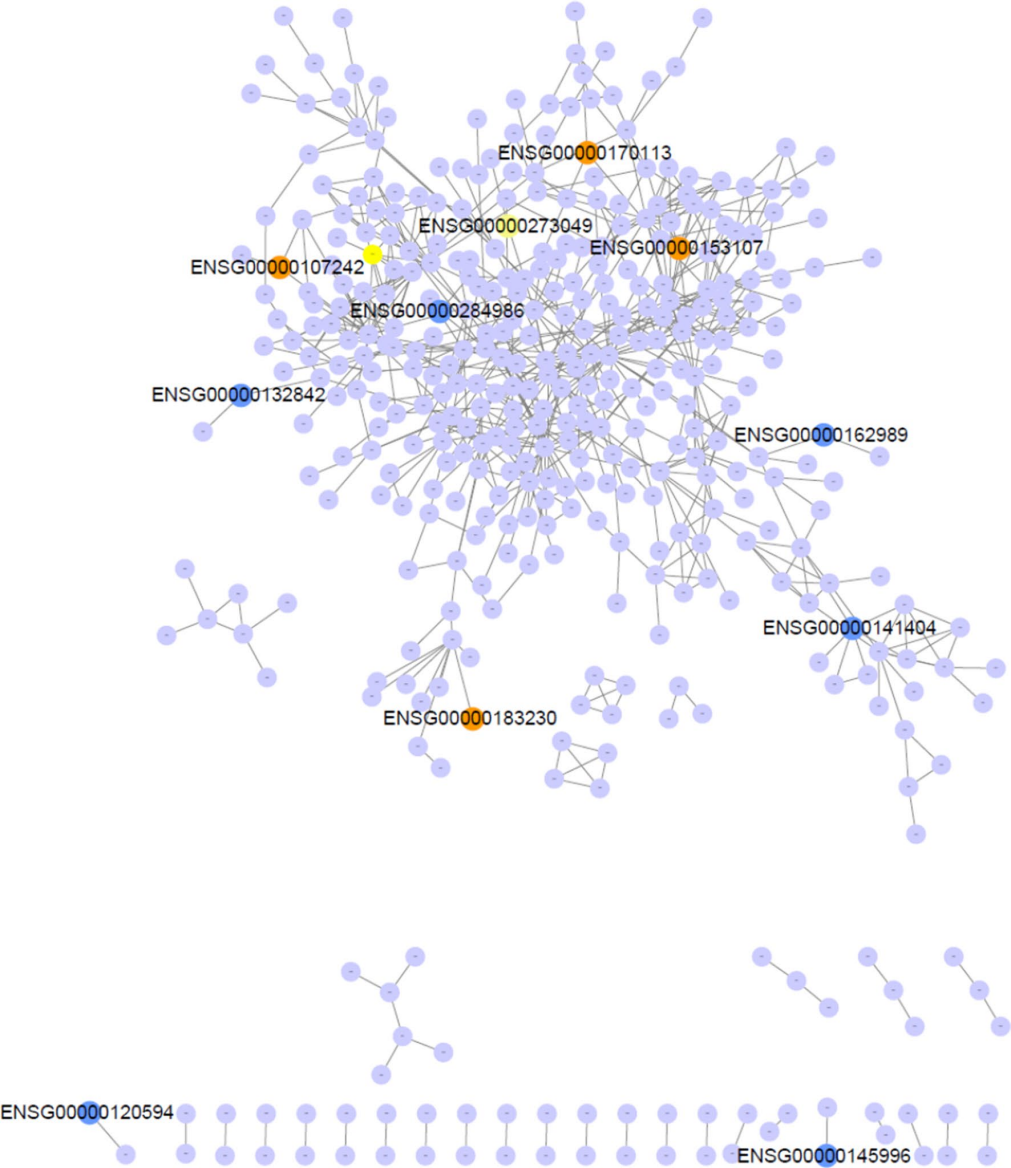
Overlap network between DEGs and DMRs. The correlation network of DEGs were generated by STRING, in which DMRs-related genes were marked in color. Hyper-, hypo- and both hyper- and hypo-methylated genes were colored in orange, blue and yellow

To explore the relationship between DMRs and DEGs, an overlap analysis was conducted. We imported the list of DEGs into the protein–protein interaction database (STRING) in order to find out the relationship between these DEGs. And then we imported the result of the interaction of DEGs into Cytoscape software to realize the visualization of the interaction network. DEGs with DMRs were colored in orange, blue and yellow for hypermethylation, hypomethylation and both, respectively (Fig. 6). Data used for the interaction network was shown in Additional file 1: Table S1.

### Quantitative real-time PCR (qRT-PCR) validation for DEGs

**Fig. 7:**
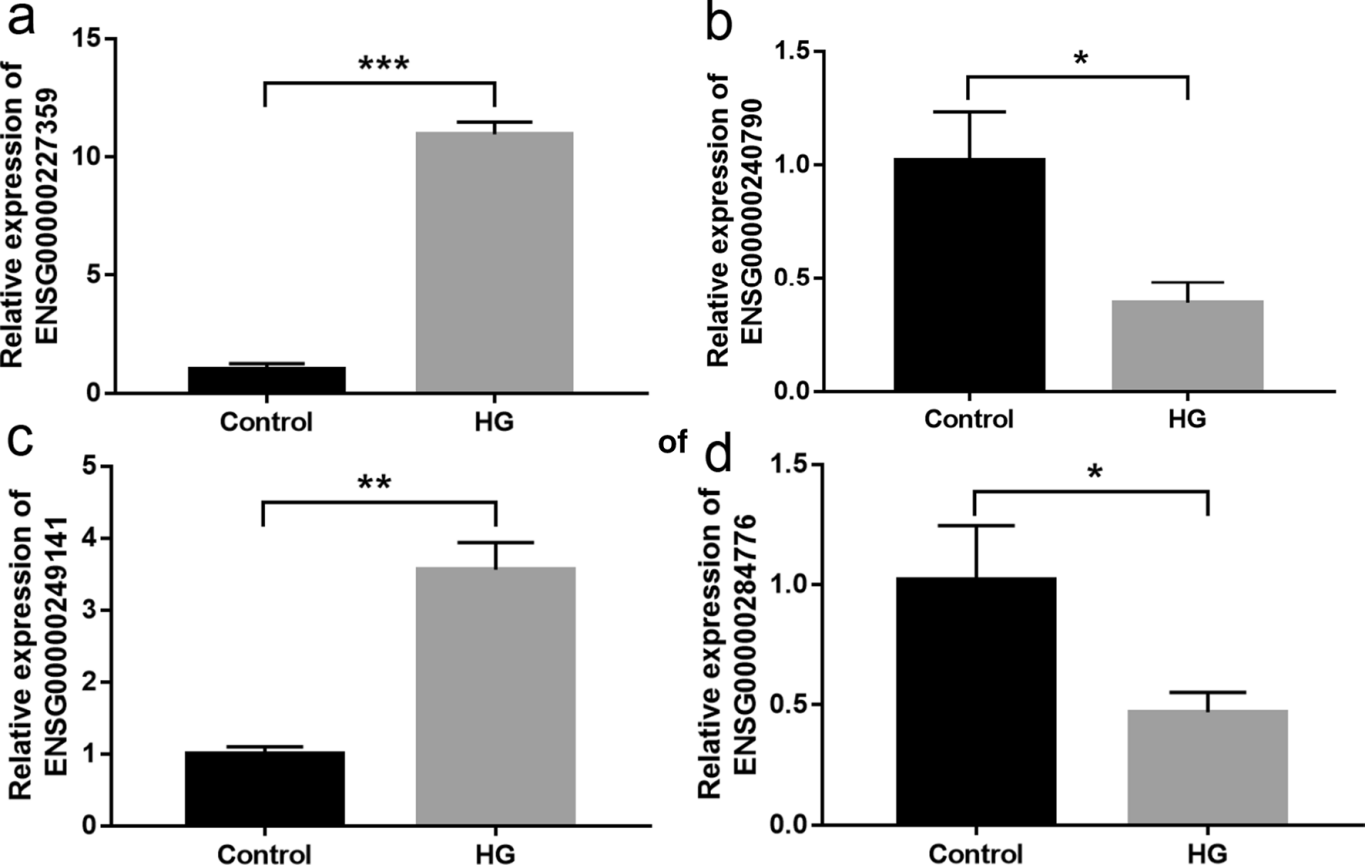
Quantitative real-time PCR validation for DEGs. ENSG00000227359 (**a**) and ENSG00000249141 (**c**) were upregulated, ENSG00000240790 (**b**) and ENSG00000284776 (**d**) were downregulated in Nthy-ori3-1 cells incubated in high-glucose medium. Data are presented as mean ± SD. *P < 0.05, **P < 0.01, ***P < 0.001

The most upregulated and downregulated mRNAs and lncRNAs were chosen for qRT-PCR validation. ENSG00000227359 and ENSG00000249141 were upregulated in Nthy-ori3-1 cells incubated in high-glucose medium (Fig. 7a and c, *P* < 0.001 and *P* = 0.005). ENSG00000240790 and ENSG00000284776 were downregulated in Nthy-ori3-1 cells incubated in high-glucose medium (Fig. 7b, d, *P* = 0.023 and *P* = 0.040).

## Discussion

The coexistence of T2DM and hypothyroidism is not accidental. Zhu et al.'s cross-sectional study on the prevalence of abnormal thyroid function in elderly T2DM patients in China suggested that hypothyroidism was the most common type of thyroid dysfunction in T2DM patients, accounting for 14.19%. The prevalence of overt and subclinical hypothyroidism was 9.3% and 4.89%, respectively [15]. Diabetes-related hypothyroidism from three perspectives were observed: patients, experimental mice, and cells. We analyzed the clinical data of 1551 T2DM patients and found that hypothyroidism was also the most common type of thyroid dysfunction, accounting for 5.67% of T2DM patients, of which overt and subclinical hypothyroidism were 3.93% and 1.74%, respectively. In addition, high TSH and low T3 and T4 levels were detected in diabetic mice. Low T3 and T4 levels were detected in Nthy-ori3-1 cells incubated in high-glucose medium.

T2DM and hypothyroidism have similar clinical symptoms, such as fatigue, edema, paleness, and weight gain [1, 16–18]. Therefore, the symptoms of T2DM can mask the occurrence of hypothyroidism. At the same time, some anti-diabetic drugs such as sulfonylureas and thiazolidinediones may also affect thyroid function [1]. Thus, T2DM patients are likely to ignore their signs and symptoms about hypothyroidism. Hypothyroidism without proper intervention and treatment may increase the risk of diabetic complications and aggravate the course of T2DM [19, 20]. It is of great significance to explore the specific mechanism of diabetic-related hypothyroidism for deepening our understanding of the disease and optimizing the treatment plan.

DNA methylation is one of the most widely studied epigenetic mechanisms regulating gene expression and mediated by DNA methyltransferases (DNMTs), among which DNMT1 mediates hemi-methylation and DNMT3A and DNMT3B were responsible for de novo methylation [21]. DNA methylation in CpG-rich promoter regions is linked to gene repression and DNA methylation within gene bodies is associated with gene activation [22]. In a study of diabetic peripheral neuropathy (DPN), the authors found that DNA methylation and gene expression profile of sural nerves in DPN patients with high HbA1c had significant changes, suggesting that the interaction between DNA methylation and gene expression participated in the pathological mechanism in DPN [23]. In this study, RNA sequencing and reduced representation bisulfite sequencing (RRBS) were performed in Nthy-ori3-1 cells cultured in high-glucose medium and normal medium, respectively. Sequencing results revealed changes in genome-wide DNA methylation and gene expression profiles. The qRT-PCR validation of specific genes confirmed the accuracy and reliability of the sequencing results.

Researchers believed that diabetes mellitus could affect thyroid function by altering TRH and TSH levels [1, 24]. Hyperglycemia led to a decrease in the activity of thyroxine 5′ deiodinase and affected the conversion of T4 to T3, which was also one of the reasons for altering thyroid function [25, 26]. Molnar et al. reported that some inflammatory cytokines including TNF-α, IFN-γ and IL-6 were related with low level of T3, which showed that high levels of inflammatory cytokines in diabetic patients might accelerate hypothyroidism [27]. Wnt signaling pathway was found under control of DNA methylation in our study and was reported to be linked to inflammation, which suggested that DNA methylation of specific genes of Wnt signaling pathway might participated in diabetes-related hypothyroidism [28, 29]. Chen et al. confirmed that high concentration of glucose inhibited proliferation of Nthy-ori3-1 cells and arrested more thyroid cells in G0/G phase [30]. This finding suggested that the reduction of thyroid cells might be partly responsible for low thyroid hormone levels. Ras-Raf-MEK-MAPK signaling pathway was reported to be involved in numerous cellular and physiological processes essential to life including cell proliferation and cell growth [31]. The KEGG analysis of DMRs revealed that Ras and MAPK signaling pathways were dysregulated in high-glucose-cultured Nthy-ori3-1 cells. DNA methylation of these signaling pathways related to cell proliferation might be a new mechanism connecting diabetes mellitus and hypothyroidism.

## Conclusions

We observed the potential connection between diabetes mellitus and hypothyroidism. This study was the first one carrying out DNA methylation and gene expression profiles to explore epigenetic modification in diabetes-related hypothyroidism, which provided information for the detailed study of the regulation of DNA methylation in diabetes-related hypothyroidism.

## Methods

### Patients

A total of 1551 T2DM patients hospitalized in our medical center were enrolled from 1 January 2018 to 31 December 2019. 374 age-matched patents without suffering from T2DM were included in the control group. All participants were older than 18 years old and complete clinical data were available. Pregnant women and participants with a history of malignant tumor or thyroid surgery were excluded in the study. The diagnostic criteria for T2DM referred to Guidelines for the prevention and control of type 2 diabetes in China (2020 Edition) [32]. The reference intervals were 0.35–5.5 IU/mL for TSH, 2.3–4.2 pg/mL for FT3 and 0.89–1.76 ng/dL for FT4 according to the commercial kit used in our medical center. The diagnostic criteria for thyroid dysfunction were as follows: overt hypothyroidism, TSH > 5.5 IU/mL, FT3 < 2.3 pg/mL and FT4 < 0.89 ng/dL; subclinical hypothyroidism, TSH > 5.5 IU/mL with normal FT3 and FT4 values; overt hyperthyroidism, TSH < 0.35 IU/mL, FT3 > 4.2 pg/mL and FT4 > 1.76 ng/dL; overt hyperthyroidism, TSH < 0.35 IU/mL with normal FT3 and FT4 values. The study was approved by the ethics committee of Beijing Hospital (No. 2021BJYYEC-013-01).

### Animals

Eight-week-old male ob/ob mice on the C57BL/6J background were purchased form the Institute of Laboratory Animals Science, CAMS & PUMC. Five-week-old male C57BL/6J mice were obtained from the Institute of Laboratory Animals Science, CAMS & PUMC and were randomly divided into two groups. One group of C57BL/6J mice were fed with a high-fat diet for 8 weeks (D12494, 60% energy from fat). The control group of C57BL/6J mice were fed with a normal diet for 8 weeks (D12450J, 10% energy from fat). Blood was collected via the orbital vein and serum was separated after coagulation for 1 h and centrifugation. All experimental procedures were approved by the experimental animal management committee of Beijing Hospital (No. P2017108).

### Cell culture

The Nthy-ori3-1 cell line were purchased from the Stem Cell Bank/Stem Cell Core Facility and were grown in RPMI-1640 medium (Hyclone, USA) with 10% fetal bovine serum (Biological Industries, Israel) at 37 °C and 5% CO_2_. High-glucose microenvironment were induced via incubating Nthy-ori3-1 cells with 20 or 30 mM high-glucose medium.

### Enzyme-linked immunosorbent assay (ELISA)

Concentrations of T3, T4 and TSH in the culture supernatant of Nthy-ori3-1 cells and mice serum were detected using ELISA kits (Cloud-Clone Corp, China). In brief, 50 μL samples were added to each well and then 50 μL prepared detection reagent A was added immediately. After incubating for 1 h at 37 °C, the plate was discarded and washed for 3 times. 100 μL prepared detection reagent B was added and the plate was incubated for 30 min at 37 °C. After washing for total 5 times, the substrate solution was added and the samples were incubated at 37 °C until color change was observed. The stop solution was added to each well and the OD values at 450 nm were measured using a microplate reader (Synergy H1, USA).

### RNA sequencing and genome-wide methylation profiling

Total RNA and DNA were extracted from Nthy-ori3-1 cells grown in high-glucose medium (glucose: 30 mM) and normal medium (glucose: 5 mM). The purity of RNA and DNA were evaluated by the NanoPhotometer® spectrophotometer (IMPLEN, USA). RNA and DNA concentrations were measured using Qubit® DNA Assay Kit in Qubit® 2.0 Flurometer (Life Technologies, USA). The quality of RNA and DNA was assessed on the Agilent Bioanalyzer 2100 system (Agilent Technologies, USA). RNA sequencing was performed on an Illumina Hiseq 2500 platform and 125 bp paired-end reads were generated. For RRBS, the library was constructed and pair-end sequencing of sample was performed on the Illumina platform.

### Quality control and data mapping

Raw data of fastq format was firstly filtered to obtain the clean data with high quality for the following analyses. For RNA sequencing data, reference genome and gene model annotation files were downloaded from genome website directly. Index of the reference genome was built and paired-end clean reads were aligned to the reference genome using HISAT2 v2.0.4. For RRBS data, bisulfite-treated reads were aligned to a reference genome using Bismark software [33]. Clean reads and reference genome were transformed into bisulfite-converted version (C-to-T and G-to-A converted). Then, the sequence reads were compared to the normal genomic sequence and the methylation state of all cytosine positions was inferred.

### Differential expression and methylation analyses

Differential expression analysis was performed using edgeR software and genes with a *P* adjust < 0.05 were assigned as differentially expressed [34]. DMRs were identified using the DSS software [35]. According to the distribution of DMRs through the genome, we defined the genes related to DMRs as genes whose gene body regions (from transcription starting site to transcription ending site) or promoter regions (2 kb upstream from the transcription starting site) have an overlap with the DMRs.

### GO and KEGG enrichment analyses

GO enrichment analyses of differentially expressed genes, lncRNA target genes and genes related to DMRs were implemented by the GOseq R package, in which gene length bias was corrected [36]. GO terms with corrected *P* value less than 0.05 were considered as significant enrichment. KOBAS software was used to test the statistical enrichment of differentially expressed genes, lncRNA target genes and genes related to DMRs in KEGG pathways [37].

### Quantitative real-time PCR

cDNA of extracted total RNA was synthesized by reverse transcription using PrimeScript™ RT Master Mix kit (Takara, Japan). Then, TB Green® Premix Ex Taq™ II kit (Takara, Japan) was used for cDNA amplification on the Bio-Rad iQ5 real-time PCR system (Bio-Rad, USA). Specific primers used were listed as follows: ENSG00000227359, 5′-TCTTCTCAGGGCCGTAAACC-3′ (forward) and 5′-GCACACTGCCAGGAATAAAGC-3′ (reverse); ENSG00000240790, 5′-AGGAAAGGACACGTCAGCATC-3′ (forward) and 5′-GAAGGACGTCATGGGCGAG-3′ (reverse); ENSG00000249141, 5′-GGCAGAAGCCTGGAACTCTAC-3′ (forward) and 5′-CTTCTGCAGTGTCTTCTGTGG-3′ (reverse); ENSG00000284776, 5′-ATCGAAATGAGTTCACGGCCT-3′ (forward) and 5′-TGTTCCAGTTCCTTCTGCGT-3′ (reverse); GAPDH, 5′-GCACCGTCAAGGCTGAGAAC-3′ (forward) and 5′-TGGTGAAGACGCCAGTGGA-3′ (reverse). Each sample was tested in triplicate. GAPDH served as the reference gene and the relative expression of target genes were calculated using the 2^−ΔΔCT^ method.

### Statistical analysis

SPSS 25.0 software was used for statistical analyses. Kolmogorov–Smirnov test was used for verifying normal distribution. Non-normally distributed variables were compared using non-parametric Mann–Whitney test. Student’s *t* test was conducted for comparison between normally distributed variables. All experiments were performed three times and *P* < 0.05 was considered to be statistically significant.

## Supplementary Information

**Additional file 1: Table S1.** Overlap network between DEGs and DMRs.

## Data Availability

The datasets used during the current study are available from the corresponding author on reasonable request.

## Abbreviations

T2DM: type 2 diabetes mellitus
OR: odd ratio
HFD: high fat diet
DEG: differentially expressed gene
DMR: differentially methylated region
qRT-PCR: quantitative real-time PCR
DNMT: DNA methyltransferase
RRBS: reduced representation bisulfite sequencing;
ELISA: enzyme-linked immunosorbent assay
