# Supplementary material for: Genome‑wide profiling of DNA methylation and gene expression unravel the epigenetic landscape in diabetes-related hypothyroidism
Source: Clin Epigenetics. 2021 Jun 6;13:123. doi: 10.1186/s13148-021-01109-2 (PMC8182906; doi:10.1186/s13148-021-01109-2)
Supplement: Supplementary file 1 — Additional file 1: Table S1. Overlap network between DEGs and DMRs. [file 13148_2021_1109_MOESM1_ESM.docx]

Additional file1: Table S1 Overlap network between DEGs and DMRs

| Gene 1 | Gene 2 | String score |
| --- | --- | --- |
| ENSG00000001630 | ENSG00000283398 | 726 |
| ENSG00000259066 | ENSG00000024048 | 774 |
| ENSG00000017797 | ENSG00000112685 | 775 |
| ENSG00000017797 | ENSG00000119729 | 899 |
| ENSG00000017797 | ENSG00000072422 | 899 |
| ENSG00000017797 | ENSG00000285245 | 824 |
| ENSG00000017797 | ENSG00000106052 | 774 |
| ENSG00000021488 | ENSG00000107242 | 708 |
| ENSG00000285218 | ENSG00000104067 | 947 |
| ENSG00000285218 | ENSG00000171217 | 800 |
| ENSG00000071655 | ENSG00000285238 | 997 |
| ENSG00000071655 | ENSG00000196591 | 995 |
| ENSG00000069424 | ENSG00000285779 | 780 |
| ENSG00000083720 | ENSG00000113790 | 899 |
| ENSG00000086827 | ENSG00000154839 | 899 |
| ENSG00000086827 | ENSG00000156970 | 988 |
| ENSG00000086827 | ENSG00000169679 | 989 |
| ENSG00000086827 | ENSG00000153107 | 706 |
| ENSG00000086827 | ENSG00000175216 | 899 |
| ENSG00000086827 | ENSG00000254483 | 899 |
| ENSG00000086827 | ENSG00000227755 | 954 |
| ENSG00000273189 | ENSG00000122025 | 952 |
| ENSG00000273189 | ENSG00000070061 | 768 |
| ENSG00000273189 | ENSG00000285330 | 772 |
| ENSG00000099937 | ENSG00000198734 | 712 |
| ENSG00000100253 | ENSG00000275374 | 825 |
| ENSG00000100253 | ENSG00000251521 | 899 |
| ENSG00000100385 | ENSG00000115145 | 899 |
| ENSG00000100385 | ENSG00000168685 | 922 |
| ENSG00000100385 | ENSG00000179295 | 899 |
| ENSG00000100385 | ENSG00000162434 | 999 |
| ENSG00000100385 | ENSG00000115415 | 987 |
| ENSG00000092201 | ENSG00000020426 | 899 |
| ENSG00000092201 | ENSG00000110768 | 899 |
| ENSG00000092201 | ENSG00000259556 | 714 |
| ENSG00000092201 | ENSG00000009954 | 710 |
| ENSG00000092201 | ENSG00000137815 | 785 |
| ENSG00000100528 | ENSG00000143742 | 893 |
| ENSG00000101292 | ENSG00000055332 | 755 |
| ENSG00000101292 | ENSG00000147246 | 899 |
| ENSG00000101292 | ENSG00000166863 | 899 |
| ENSG00000101292 | ENSG00000257002 | 899 |
| ENSG00000101292 | ENSG00000075073 | 899 |
| ENSG00000250917 | ENSG00000151224 | 941 |
| ENSG00000104408 | ENSG00000164346 | 754 |
| ENSG00000104408 | ENSG00000181163 | 763 |
| ENSG00000104408 | ENSG00000228568 | 942 |
| ENSG00000104408 | ENSG00000197170 | 758 |
| ENSG00000104408 | ENSG00000125977 | 929 |
| ENSG00000104408 | ENSG00000256552 | 942 |
| ENSG00000268083 | ENSG00000125870 | 899 |
| ENSG00000268083 | ENSG00000168438 | 899 |
| ENSG00000268083 | ENSG00000265241 | 899 |
| ENSG00000268083 | ENSG00000135829 | 907 |
| ENSG00000104976 | ENSG00000285446 | 741 |
| ENSG00000232626 | ENSG00000080824 | 977 |
| ENSG00000232626 | ENSG00000198668 | 857 |
| ENSG00000021461 | ENSG00000138115 | 913 |
| ENSG00000105829 | ENSG00000138802 | 751 |
| ENSG00000105829 | ENSG00000274423 | 987 |
| ENSG00000095585 | ENSG00000224003 | 900 |
| ENSG00000095585 | ENSG00000147010 | 961 |
| ENSG00000108878 | ENSG00000113319 | 800 |
| ENSG00000285043 | ENSG00000235076 | 971 |
| ENSG00000285043 | ENSG00000149925 | 809 |
| ENSG00000285043 | ENSG00000278419 | 899 |
| ENSG00000250412 | ENSG00000036257 | 938 |
| ENSG00000066336 | ENSG00000122025 | 989 |
| ENSG00000066336 | ENSG00000124789 | 873 |
| ENSG00000066336 | ENSG00000168685 | 948 |
| ENSG00000066336 | ENSG00000102024 | 764 |
| ENSG00000066336 | ENSG00000119535 | 907 |
| ENSG00000066336 | ENSG00000189403 | 866 |
| ENSG00000066336 | ENSG00000285625 | 852 |
| ENSG00000066336 | ENSG00000118260 | 740 |
| ENSG00000111247 | ENSG00000112742 | 853 |
| ENSG00000111247 | ENSG00000285920 | 876 |
| ENSG00000111247 | ENSG00000156970 | 868 |
| ENSG00000111247 | ENSG00000165304 | 860 |
| ENSG00000111247 | ENSG00000138180 | 882 |
| ENSG00000111247 | ENSG00000072571 | 795 |
| ENSG00000235076 | ENSG00000241837 | 805 |
| ENSG00000235076 | ENSG00000275374 | 700 |
| ENSG00000235076 | ENSG00000213131 | 798 |
| ENSG00000235076 | ENSG00000149925 | 995 |
| ENSG00000235076 | ENSG00000144381 | 952 |
| ENSG00000235076 | ENSG00000181555 | 835 |
| ENSG00000235076 | ENSG00000111716 | 868 |
| ENSG00000112685 | ENSG00000119729 | 899 |
| ENSG00000112742 | ENSG00000285920 | 893 |
| ENSG00000112742 | ENSG00000156970 | 941 |
| ENSG00000112742 | ENSG00000165304 | 883 |
| ENSG00000112742 | ENSG00000169679 | 932 |
| ENSG00000112742 | ENSG00000138180 | 885 |
| ENSG00000112742 | ENSG00000090889 | 845 |
| ENSG00000112742 | ENSG00000072571 | 871 |
| ENSG00000112742 | ENSG00000227755 | 912 |
| ENSG00000113569 | ENSG00000205578 | 924 |
| ENSG00000113569 | ENSG00000124789 | 963 |
| ENSG00000113569 | ENSG00000155561 | 977 |
| ENSG00000113569 | ENSG00000108424 | 912 |
| ENSG00000113569 | ENSG00000168438 | 899 |
| ENSG00000113569 | ENSG00000265241 | 901 |
| ENSG00000113643 | ENSG00000115866 | 946 |
| ENSG00000113643 | ENSG00000077092 | 946 |
| ENSG00000113643 | ENSG00000136628 | 983 |
| ENSG00000113643 | ENSG00000133706 | 977 |
| ENSG00000113790 | ENSG00000133835 | 884 |
| ENSG00000113790 | ENSG00000196177 | 916 |
| ENSG00000113790 | ENSG00000255967 | 914 |
| ENSG00000114450 | ENSG00000065135 | 800 |
| ENSG00000055332 | ENSG00000181163 | 884 |
| ENSG00000055332 | ENSG00000080824 | 855 |
| ENSG00000055332 | ENSG00000179295 | 943 |
| ENSG00000055332 | ENSG00000162434 | 713 |
| ENSG00000055332 | ENSG00000260886 | 898 |
| ENSG00000055332 | ENSG00000115415 | 984 |
| ENSG00000055332 | ENSG00000118217 | 833 |
| ENSG00000055332 | ENSG00000125977 | 858 |
| ENSG00000055332 | ENSG00000102580 | 997 |
| ENSG00000055332 | ENSG00000254905 | 793 |
| ENSG00000055332 | ENSG00000144895 | 976 |
| ENSG00000079785 | ENSG00000134440 | 702 |
| ENSG00000079785 | ENSG00000154174 | 728 |
| ENSG00000079785 | ENSG00000197170 | 781 |
| ENSG00000079785 | ENSG00000124767 | 704 |
| ENSG00000079785 | ENSG00000173039 | 933 |
| ENSG00000079785 | ENSG00000115233 | 798 |
| ENSG00000095002 | ENSG00000116062 | 999 |
| ENSG00000095002 | ENSG00000035928 | 946 |
| ENSG00000095002 | ENSG00000174371 | 999 |
| ENSG00000095002 | ENSG00000079246 | 710 |
| ENSG00000095002 | ENSG00000197299 | 765 |
| ENSG00000095002 | ENSG00000171862 | 878 |
| ENSG00000023228 | ENSG00000164258 | 998 |
| ENSG00000023228 | ENSG00000248648 | 899 |
| ENSG00000023228 | ENSG00000258674 | 899 |
| ENSG00000115816 | ENSG00000044524 | 700 |
| ENSG00000115816 | ENSG00000067955 | 857 |
| ENSG00000115942 | ENSG00000135336 | 999 |
| ENSG00000115942 | ENSG00000115947 | 999 |
| ENSG00000115942 | ENSG00000092853 | 899 |
| ENSG00000115942 | ENSG00000285625 | 906 |
| ENSG00000115942 | ENSG00000065328 | 998 |
| ENSG00000115942 | ENSG00000283544 | 904 |
| ENSG00000116062 | ENSG00000035928 | 929 |
| ENSG00000116062 | ENSG00000174371 | 953 |
| ENSG00000116062 | ENSG00000197299 | 703 |
| ENSG00000116062 | ENSG00000171862 | 885 |
| ENSG00000117281 | ENSG00000030304 | 834 |
| ENSG00000118985 | ENSG00000144218 | 811 |
| ENSG00000119729 | ENSG00000137962 | 899 |
| ENSG00000119729 | ENSG00000113319 | 899 |
| ENSG00000119729 | ENSG00000072422 | 908 |
| ENSG00000119729 | ENSG00000079482 | 925 |
| ENSG00000119729 | ENSG00000057608 | 899 |
| ENSG00000121075 | ENSG00000273049 | 943 |
| ENSG00000245648 | ENSG00000164520 | 993 |
| ENSG00000122025 | ENSG00000115008 | 800 |
| ENSG00000122025 | ENSG00000181163 | 897 |
| ENSG00000122025 | ENSG00000168685 | 949 |
| ENSG00000122025 | ENSG00000080824 | 804 |
| ENSG00000122025 | ENSG00000179295 | 907 |
| ENSG00000122025 | ENSG00000117400 | 730 |
| ENSG00000106546 | ENSG00000084676 | 802 |
| ENSG00000106546 | ENSG00000080824 | 992 |
| ENSG00000106546 | ENSG00000115415 | 822 |
| ENSG00000106546 | ENSG00000173039 | 933 |
| ENSG00000106546 | ENSG00000181555 | 949 |
| ENSG00000241104 | ENSG00000150093 | 708 |
| ENSG00000125870 | ENSG00000168438 | 899 |
| ENSG00000125870 | ENSG00000265241 | 906 |
| ENSG00000125870 | ENSG00000135829 | 953 |
| ENSG00000126775 | ENSG00000196455 | 956 |
| ENSG00000128595 | ENSG00000104067 | 750 |
| ENSG00000129083 | ENSG00000138802 | 707 |
| ENSG00000264958 | ENSG00000138115 | 900 |
| ENSG00000130287 | ENSG00000106819 | 919 |
| ENSG00000130287 | ENSG00000189403 | 742 |
| ENSG00000130741 | ENSG00000125977 | 991 |
| ENSG00000131725 | ENSG00000271092 | 718 |
| ENSG00000173473 | ENSG00000084676 | 903 |
| ENSG00000173473 | ENSG00000009954 | 959 |
| ENSG00000173473 | ENSG00000163939 | 796 |
| ENSG00000173473 | ENSG00000077097 | 899 |
| ENSG00000173473 | ENSG00000077080 | 982 |
| ENSG00000132842 | ENSG00000141367 | 743 |
| ENSG00000132842 | ENSG00000185009 | 895 |
| ENSG00000064726 | ENSG00000198900 | 922 |
| ENSG00000133704 | ENSG00000108424 | 704 |
| ENSG00000134440 | ENSG00000143742 | 722 |
| ENSG00000134440 | ENSG00000120705 | 750 |
| ENSG00000134440 | ENSG00000136628 | 773 |
| ENSG00000134440 | ENSG00000133706 | 782 |
| ENSG00000134440 | ENSG00000115233 | 763 |
| ENSG00000135336 | ENSG00000115947 | 999 |
| ENSG00000135336 | ENSG00000092853 | 899 |
| ENSG00000135336 | ENSG00000285625 | 901 |
| ENSG00000135336 | ENSG00000065328 | 991 |
| ENSG00000135336 | ENSG00000283544 | 899 |
| ENSG00000136731 | ENSG00000232433 | 792 |
| ENSG00000136813 | ENSG00000173692 | 901 |
| ENSG00000136813 | ENSG00000267430 | 900 |
| ENSG00000136813 | ENSG00000197170 | 901 |
| ENSG00000136813 | ENSG00000115233 | 971 |
| ENSG00000137575 | ENSG00000164418 | 894 |
| ENSG00000285920 | ENSG00000156970 | 874 |
| ENSG00000285920 | ENSG00000165304 | 871 |
| ENSG00000285920 | ENSG00000146918 | 789 |
| ENSG00000285920 | ENSG00000138180 | 886 |
| ENSG00000285920 | ENSG00000090889 | 890 |
| ENSG00000285920 | ENSG00000072571 | 942 |
| ENSG00000137807 | ENSG00000213131 | 889 |
| ENSG00000137807 | ENSG00000138180 | 970 |
| ENSG00000137876 | ENSG00000164346 | 907 |
| ENSG00000137962 | ENSG00000152580 | 712 |
| ENSG00000137962 | ENSG00000072422 | 899 |
| ENSG00000138193 | ENSG00000107242 | 899 |
| ENSG00000138193 | ENSG00000171862 | 899 |
| ENSG00000138193 | ENSG00000107789 | 899 |
| ENSG00000138193 | ENSG00000251521 | 899 |
| ENSG00000020426 | ENSG00000138785 | 899 |
| ENSG00000020426 | ENSG00000110768 | 974 |
| ENSG00000020426 | ENSG00000259556 | 899 |
| ENSG00000020426 | ENSG00000178913 | 899 |
| ENSG00000020426 | ENSG00000143493 | 899 |
| ENSG00000020426 | ENSG00000111880 | 899 |
| ENSG00000020426 | ENSG00000070061 | 899 |
| ENSG00000100764 | ENSG00000101557 | 972 |
| ENSG00000100764 | ENSG00000156970 | 899 |
| ENSG00000100764 | ENSG00000173692 | 999 |
| ENSG00000100764 | ENSG00000267430 | 999 |
| ENSG00000100764 | ENSG00000153107 | 899 |
| ENSG00000100764 | ENSG00000197170 | 966 |
| ENSG00000100764 | ENSG00000115233 | 998 |
| ENSG00000035928 | ENSG00000077514 | 973 |
| ENSG00000035928 | ENSG00000062650 | 892 |
| ENSG00000035928 | ENSG00000256514 | 937 |
| ENSG00000035928 | ENSG00000174371 | 770 |
| ENSG00000035928 | ENSG00000197299 | 765 |
| ENSG00000035928 | ENSG00000285625 | 922 |
| ENSG00000035928 | ENSG00000173039 | 925 |
| ENSG00000035928 | ENSG00000228716 | 780 |
| ENSG00000035928 | ENSG00000283544 | 906 |
| ENSG00000065150 | ENSG00000124789 | 851 |
| ENSG00000065150 | ENSG00000155561 | 915 |
| ENSG00000065150 | ENSG00000108424 | 947 |
| ENSG00000065150 | ENSG00000184575 | 925 |
| ENSG00000065150 | ENSG00000205339 | 988 |
| ENSG00000065150 | ENSG00000227755 | 899 |
| ENSG00000083093 | ENSG00000197299 | 741 |
| ENSG00000083093 | ENSG00000171862 | 754 |
| ENSG00000101557 | ENSG00000173692 | 985 |
| ENSG00000101557 | ENSG00000267430 | 970 |
| ENSG00000101557 | ENSG00000197170 | 988 |
| ENSG00000101557 | ENSG00000115233 | 994 |
| ENSG00000261418 | ENSG00000198211 | 796 |
| ENSG00000124789 | ENSG00000155561 | 966 |
| ENSG00000124789 | ENSG00000108424 | 998 |
| ENSG00000124789 | ENSG00000168438 | 899 |
| ENSG00000124789 | ENSG00000184575 | 799 |
| ENSG00000124789 | ENSG00000265241 | 899 |
| ENSG00000108395 | ENSG00000065328 | 709 |
| ENSG00000116489 | ENSG00000164961 | 899 |
| ENSG00000115008 | ENSG00000196611 | 948 |
| ENSG00000115008 | ENSG00000115415 | 722 |
| ENSG00000141979 | ENSG00000125686 | 983 |
| ENSG00000141979 | ENSG00000054118 | 899 |
| ENSG00000141979 | ENSG00000130772 | 923 |
| ENSG00000077514 | ENSG00000256514 | 995 |
| ENSG00000077514 | ENSG00000285625 | 940 |
| ENSG00000077514 | ENSG00000283544 | 899 |
| ENSG00000128829 | ENSG00000080824 | 835 |
| ENSG00000128829 | ENSG00000118217 | 739 |
| ENSG00000115145 | ENSG00000162434 | 982 |
| ENSG00000115145 | ENSG00000147010 | 899 |
| ENSG00000110367 | ENSG00000235081 | 736 |
| ENSG00000115866 | ENSG00000136628 | 949 |
| ENSG00000115866 | ENSG00000133706 | 915 |
| ENSG00000115947 | ENSG00000092853 | 899 |
| ENSG00000115947 | ENSG00000079246 | 702 |
| ENSG00000115947 | ENSG00000285625 | 941 |
| ENSG00000115947 | ENSG00000065328 | 991 |
| ENSG00000115947 | ENSG00000283544 | 900 |
| ENSG00000128059 | ENSG00000035687 | 905 |
| ENSG00000128059 | ENSG00000101911 | 945 |
| ENSG00000128059 | ENSG00000128050 | 996 |
| ENSG00000036257 | ENSG00000072422 | 848 |
| ENSG00000117222 | ENSG00000178913 | 899 |
| ENSG00000073282 | ENSG00000067369 | 782 |
| ENSG00000144668 | ENSG00000150093 | 999 |
| ENSG00000038427 | ENSG00000106819 | 896 |
| ENSG00000038427 | ENSG00000139684 | 784 |
| ENSG00000038427 | ENSG00000284986 | 865 |
| ENSG00000113319 | ENSG00000148408 | 800 |
| ENSG00000113319 | ENSG00000072422 | 899 |
| ENSG00000113319 | ENSG00000145349 | 899 |
| ENSG00000113319 | ENSG00000198668 | 792 |
| ENSG00000138785 | ENSG00000110768 | 899 |
| ENSG00000138785 | ENSG00000259556 | 899 |
| ENSG00000138785 | ENSG00000178913 | 899 |
| ENSG00000138785 | ENSG00000143493 | 899 |
| ENSG00000138785 | ENSG00000070061 | 899 |
| ENSG00000138802 | ENSG00000178913 | 728 |
| ENSG00000138802 | ENSG00000270757 | 714 |
| ENSG00000138802 | ENSG00000136758 | 833 |
| ENSG00000107242 | ENSG00000171862 | 899 |
| ENSG00000082556 | ENSG00000253690 | 995 |
| ENSG00000082556 | ENSG00000135898 | 899 |
| ENSG00000110768 | ENSG00000259556 | 899 |
| ENSG00000110768 | ENSG00000178913 | 899 |
| ENSG00000110768 | ENSG00000143493 | 899 |
| ENSG00000110768 | ENSG00000111880 | 899 |
| ENSG00000110768 | ENSG00000070061 | 899 |
| ENSG00000140379 | ENSG00000173039 | 778 |
| ENSG00000141367 | ENSG00000157404 | 709 |
| ENSG00000141367 | ENSG00000166747 | 912 |
| ENSG00000133026 | ENSG00000261217 | 825 |
| ENSG00000255730 | ENSG00000196177 | 905 |
| ENSG00000144407 | ENSG00000198211 | 899 |
| ENSG00000144407 | ENSG00000128271 | 899 |
| ENSG00000144407 | ENSG00000148680 | 899 |
| ENSG00000144407 | ENSG00000175868 | 899 |
| ENSG00000144566 | ENSG00000057608 | 797 |
| ENSG00000144566 | ENSG00000166747 | 750 |
| ENSG00000144579 | ENSG00000197971 | 720 |
| ENSG00000144671 | ENSG00000172939 | 714 |
| ENSG00000145996 | ENSG00000148737 | 855 |
| ENSG00000146731 | ENSG00000218226 | 710 |
| ENSG00000146731 | ENSG00000270757 | 857 |
| ENSG00000280828 | ENSG00000197971 | 750 |
| ENSG00000147246 | ENSG00000166863 | 899 |
| ENSG00000147246 | ENSG00000257002 | 914 |
| ENSG00000147246 | ENSG00000075073 | 899 |
| ENSG00000088387 | ENSG00000155850 | 736 |
| ENSG00000149016 | ENSG00000238172 | 833 |
| ENSG00000149016 | ENSG00000237169 | 855 |
| ENSG00000149016 | ENSG00000251354 | 895 |
| ENSG00000149021 | ENSG00000082175 | 778 |
| ENSG00000149021 | ENSG00000251201 | 730 |
| ENSG00000149218 | ENSG00000106780 | 714 |
| ENSG00000149218 | ENSG00000136193 | 706 |
| ENSG00000104067 | ENSG00000270149 | 999 |
| ENSG00000104067 | ENSG00000180353 | 800 |
| ENSG00000104067 | ENSG00000119326 | 767 |
| ENSG00000104067 | ENSG00000171217 | 809 |
| ENSG00000104067 | ENSG00000183230 | 950 |
| ENSG00000104067 | ENSG00000238133 | 800 |
| ENSG00000104067 | ENSG00000254732 | 986 |
| ENSG00000152822 | ENSG00000186063 | 786 |
| ENSG00000152822 | ENSG00000145349 | 817 |
| ENSG00000152822 | ENSG00000065135 | 800 |
| ENSG00000152822 | ENSG00000124493 | 908 |
| ENSG00000275835 | ENSG00000170113 | 965 |
| ENSG00000275835 | ENSG00000175216 | 910 |
| ENSG00000275835 | ENSG00000251143 | 927 |
| ENSG00000275835 | ENSG00000113712 | 899 |
| ENSG00000275835 | ENSG00000077380 | 899 |
| ENSG00000153827 | ENSG00000181163 | 749 |
| ENSG00000154174 | ENSG00000080824 | 807 |
| ENSG00000154174 | ENSG00000197170 | 746 |
| ENSG00000154839 | ENSG00000156970 | 925 |
| ENSG00000154839 | ENSG00000169679 | 956 |
| ENSG00000154839 | ENSG00000175216 | 899 |
| ENSG00000154839 | ENSG00000198668 | 899 |
| ENSG00000154839 | ENSG00000251143 | 899 |
| ENSG00000154839 | ENSG00000160783 | 899 |
| ENSG00000154839 | ENSG00000090889 | 920 |
| ENSG00000154839 | ENSG00000102699 | 899 |
| ENSG00000154839 | ENSG00000227755 | 899 |
| ENSG00000155561 | ENSG00000108424 | 913 |
| ENSG00000155561 | ENSG00000168438 | 899 |
| ENSG00000155561 | ENSG00000184575 | 903 |
| ENSG00000155561 | ENSG00000265241 | 899 |
| ENSG00000155561 | ENSG00000205339 | 966 |
| ENSG00000155561 | ENSG00000227755 | 899 |
| ENSG00000156970 | ENSG00000165304 | 900 |
| ENSG00000156970 | ENSG00000169679 | 999 |
| ENSG00000156970 | ENSG00000173692 | 899 |
| ENSG00000156970 | ENSG00000267430 | 922 |
| ENSG00000156970 | ENSG00000153107 | 999 |
| ENSG00000156970 | ENSG00000175216 | 949 |
| ENSG00000156970 | ENSG00000197170 | 899 |
| ENSG00000156970 | ENSG00000146918 | 753 |
| ENSG00000156970 | ENSG00000143199 | 784 |
| ENSG00000156970 | ENSG00000160783 | 899 |
| ENSG00000156970 | ENSG00000138180 | 937 |
| ENSG00000156970 | ENSG00000090889 | 884 |
| ENSG00000156970 | ENSG00000254483 | 937 |
| ENSG00000156970 | ENSG00000072571 | 910 |
| ENSG00000156970 | ENSG00000227755 | 978 |
| ENSG00000156970 | ENSG00000115233 | 913 |
| ENSG00000157404 | ENSG00000181163 | 744 |
| ENSG00000157404 | ENSG00000168685 | 801 |
| ENSG00000157404 | ENSG00000179295 | 989 |
| ENSG00000157404 | ENSG00000115415 | 971 |
| ENSG00000157404 | ENSG00000117400 | 838 |
| ENSG00000157404 | ENSG00000078747 | 800 |
| ENSG00000157404 | ENSG00000150093 | 764 |
| ENSG00000157404 | ENSG00000147010 | 902 |
| ENSG00000108424 | ENSG00000184575 | 910 |
| ENSG00000108424 | ENSG00000186432 | 962 |
| ENSG00000108424 | ENSG00000205339 | 984 |
| ENSG00000108424 | ENSG00000227755 | 899 |
| ENSG00000243646 | ENSG00000162434 | 968 |
| ENSG00000243646 | ENSG00000268510 | 996 |
| ENSG00000243646 | ENSG00000115415 | 964 |
| ENSG00000241837 | ENSG00000230585 | 770 |
| ENSG00000241837 | ENSG00000144381 | 740 |
| ENSG00000241837 | ENSG00000198899 | 999 |
| ENSG00000257524 | ENSG00000157350 | 905 |
| ENSG00000162433 | ENSG00000167325 | 899 |
| ENSG00000162433 | ENSG00000256879 | 899 |
| ENSG00000162989 | ENSG00000228620 | 910 |
| ENSG00000162989 | ENSG00000117152 | 720 |
| ENSG00000163840 | ENSG00000067369 | 728 |
| ENSG00000163993 | ENSG00000254483 | 740 |
| ENSG00000163993 | ENSG00000118260 | 819 |
| ENSG00000164258 | ENSG00000248648 | 920 |
| ENSG00000164258 | ENSG00000258674 | 899 |
| ENSG00000164346 | ENSG00000181163 | 794 |
| ENSG00000164346 | ENSG00000198301 | 794 |
| ENSG00000181163 | ENSG00000126785 | 799 |
| ENSG00000181163 | ENSG00000144381 | 854 |
| ENSG00000181163 | ENSG00000179295 | 829 |
| ENSG00000181163 | ENSG00000251143 | 991 |
| ENSG00000181163 | ENSG00000198900 | 896 |
| ENSG00000181163 | ENSG00000166197 | 800 |
| ENSG00000181163 | ENSG00000077097 | 793 |
| ENSG00000181163 | ENSG00000067955 | 720 |
| ENSG00000164597 | ENSG00000260136 | 997 |
| ENSG00000154188 | ENSG00000091879 | 961 |
| ENSG00000154188 | ENSG00000179295 | 922 |
| ENSG00000154188 | ENSG00000150093 | 914 |
| ENSG00000154188 | ENSG00000134954 | 908 |
| ENSG00000154188 | ENSG00000173039 | 899 |
| ENSG00000164951 | ENSG00000171862 | 764 |
| ENSG00000165304 | ENSG00000146918 | 702 |
| ENSG00000165304 | ENSG00000138180 | 900 |
| ENSG00000165304 | ENSG00000090889 | 891 |
| ENSG00000165304 | ENSG00000072571 | 854 |
| ENSG00000165449 | ENSG00000079691 | 738 |
| ENSG00000165449 | ENSG00000215859 | 746 |
| ENSG00000254641 | ENSG00000171862 | 774 |
| ENSG00000254641 | ENSG00000150093 | 995 |
| ENSG00000259556 | ENSG00000178913 | 899 |
| ENSG00000259556 | ENSG00000143493 | 899 |
| ENSG00000259556 | ENSG00000070061 | 916 |
| ENSG00000259556 | ENSG00000137815 | 955 |
| ENSG00000166863 | ENSG00000257002 | 900 |
| ENSG00000166863 | ENSG00000175868 | 830 |
| ENSG00000166863 | ENSG00000075073 | 996 |
| ENSG00000230585 | ENSG00000144381 | 957 |
| ENSG00000230585 | ENSG00000151224 | 820 |
| ENSG00000125686 | ENSG00000054118 | 953 |
| ENSG00000125686 | ENSG00000130772 | 961 |
| ENSG00000167325 | ENSG00000285625 | 713 |
| ENSG00000167325 | ENSG00000171862 | 840 |
| ENSG00000167769 | ENSG00000255319 | 925 |
| ENSG00000253690 | ENSG00000175868 | 714 |
| ENSG00000253690 | ENSG00000135898 | 899 |
| ENSG00000169679 | ENSG00000175216 | 899 |
| ENSG00000169679 | ENSG00000143199 | 758 |
| ENSG00000169679 | ENSG00000160783 | 998 |
| ENSG00000169679 | ENSG00000254483 | 899 |
| ENSG00000169679 | ENSG00000072571 | 792 |
| ENSG00000169679 | ENSG00000227755 | 952 |
| ENSG00000171940 | ENSG00000118260 | 826 |
| ENSG00000168438 | ENSG00000265241 | 899 |
| ENSG00000168438 | ENSG00000135829 | 899 |
| ENSG00000103044 | ENSG00000072571 | 758 |
| ENSG00000154719 | ENSG00000228568 | 734 |
| ENSG00000168685 | ENSG00000224003 | 845 |
| ENSG00000168685 | ENSG00000119535 | 707 |
| ENSG00000168685 | ENSG00000162434 | 994 |
| ENSG00000168685 | ENSG00000134954 | 818 |
| ENSG00000128641 | ENSG00000198668 | 843 |
| ENSG00000257002 | ENSG00000141404 | 866 |
| ENSG00000257002 | ENSG00000172380 | 800 |
| ENSG00000257002 | ENSG00000075073 | 910 |
| ENSG00000257002 | ENSG00000117152 | 743 |
| ENSG00000273049 | ENSG00000144381 | 899 |
| ENSG00000088053 | ENSG00000198668 | 958 |
| ENSG00000173692 | ENSG00000267430 | 999 |
| ENSG00000173692 | ENSG00000153107 | 899 |
| ENSG00000173692 | ENSG00000197170 | 977 |
| ENSG00000173692 | ENSG00000115233 | 999 |
| ENSG00000213131 | ENSG00000113712 | 899 |
| ENSG00000213131 | ENSG00000138071 | 770 |
| ENSG00000213131 | ENSG00000187686 | 909 |
| ENSG00000175334 | ENSG00000094916 | 723 |
| ENSG00000175334 | ENSG00000009954 | 875 |
| ENSG00000175334 | ENSG00000077080 | 981 |
| ENSG00000267430 | ENSG00000153107 | 899 |
| ENSG00000267430 | ENSG00000197170 | 953 |
| ENSG00000267430 | ENSG00000108946 | 772 |
| ENSG00000267430 | ENSG00000115233 | 975 |
| ENSG00000010282 | ENSG00000178104 | 700 |
| ENSG00000257735 | ENSG00000254444 | 899 |
| ENSG00000257735 | ENSG00000141404 | 899 |
| ENSG00000256514 | ENSG00000285625 | 919 |
| ENSG00000256514 | ENSG00000283544 | 899 |
| ENSG00000174371 | ENSG00000197299 | 810 |
| ENSG00000173402 | ENSG00000106819 | 943 |
| ENSG00000173402 | ENSG00000198947 | 999 |
| ENSG00000173402 | ENSG00000030304 | 809 |
| ENSG00000178913 | ENSG00000143742 | 804 |
| ENSG00000178913 | ENSG00000143493 | 899 |
| ENSG00000178913 | ENSG00000070061 | 899 |
| ENSG00000092853 | ENSG00000065328 | 899 |
| ENSG00000070756 | ENSG00000110321 | 767 |
| ENSG00000070756 | ENSG00000120705 | 913 |
| ENSG00000253729 | ENSG00000138303 | 899 |
| ENSG00000253729 | ENSG00000101773 | 881 |
| ENSG00000253729 | ENSG00000082175 | 800 |
| ENSG00000253729 | ENSG00000079246 | 999 |
| ENSG00000253729 | ENSG00000189403 | 826 |
| ENSG00000253729 | ENSG00000198900 | 854 |
| ENSG00000253729 | ENSG00000160783 | 899 |
| ENSG00000253729 | ENSG00000067369 | 820 |
| ENSG00000253729 | ENSG00000173039 | 911 |
| ENSG00000253729 | ENSG00000251201 | 853 |
| ENSG00000091879 | ENSG00000134954 | 990 |
| ENSG00000091879 | ENSG00000181555 | 762 |
| ENSG00000143742 | ENSG00000136758 | 708 |
| ENSG00000143742 | ENSG00000108946 | 720 |
| ENSG00000143742 | ENSG00000152558 | 704 |
| ENSG00000177103 | ENSG00000120594 | 704 |
| ENSG00000270757 | ENSG00000197170 | 746 |
| ENSG00000270757 | ENSG00000102230 | 857 |
| ENSG00000102024 | ENSG00000139684 | 774 |
| ENSG00000178691 | ENSG00000196591 | 902 |
| ENSG00000126785 | ENSG00000197971 | 778 |
| ENSG00000083535 | ENSG00000082175 | 824 |
| ENSG00000160185 | ENSG00000236444 | 749 |
| ENSG00000164961 | ENSG00000170113 | 839 |
| ENSG00000136758 | ENSG00000197170 | 702 |
| ENSG00000228568 | ENSG00000238172 | 892 |
| ENSG00000228568 | ENSG00000120705 | 911 |
| ENSG00000228568 | ENSG00000237169 | 992 |
| ENSG00000228568 | ENSG00000251354 | 844 |
| ENSG00000228568 | ENSG00000125977 | 901 |
| ENSG00000228568 | ENSG00000256552 | 995 |
| ENSG00000170606 | ENSG00000080824 | 860 |
| ENSG00000170606 | ENSG00000144381 | 764 |
| ENSG00000170606 | ENSG00000086061 | 813 |
| ENSG00000225101 | ENSG00000141404 | 800 |
| ENSG00000198211 | ENSG00000128271 | 899 |
| ENSG00000198211 | ENSG00000148680 | 899 |
| ENSG00000198211 | ENSG00000175868 | 899 |
| ENSG00000198211 | ENSG00000107165 | 946 |
| ENSG00000119326 | ENSG00000170776 | 970 |
| ENSG00000138303 | ENSG00000160783 | 899 |
| ENSG00000138303 | ENSG00000173039 | 954 |
| ENSG00000084676 | ENSG00000082175 | 981 |
| ENSG00000084676 | ENSG00000077092 | 847 |
| ENSG00000084676 | ENSG00000236444 | 876 |
| ENSG00000084676 | ENSG00000118260 | 885 |
| ENSG00000082212 | ENSG00000144381 | 716 |
| ENSG00000254444 | ENSG00000141404 | 899 |
| ENSG00000196611 | ENSG00000106819 | 728 |
| ENSG00000196611 | ENSG00000134954 | 954 |
| ENSG00000224003 | ENSG00000044524 | 899 |
| ENSG00000224003 | ENSG00000179295 | 903 |
| ENSG00000224003 | ENSG00000254732 | 907 |
| ENSG00000082175 | ENSG00000080824 | 966 |
| ENSG00000082175 | ENSG00000285622 | 851 |
| ENSG00000258728 | ENSG00000162434 | 869 |
| ENSG00000184575 | ENSG00000205339 | 980 |
| ENSG00000184575 | ENSG00000227755 | 899 |
| ENSG00000079246 | ENSG00000067369 | 732 |
| ENSG00000079246 | ENSG00000077097 | 866 |
| ENSG00000285446 | ENSG00000118260 | 842 |
| ENSG00000077092 | ENSG00000198963 | 903 |
| ENSG00000265241 | ENSG00000135829 | 901 |
| ENSG00000265241 | ENSG00000170473 | 969 |
| ENSG00000176358 | ENSG00000075073 | 931 |
| ENSG00000141404 | ENSG00000128271 | 800 |
| ENSG00000141404 | ENSG00000148680 | 800 |
| ENSG00000186432 | ENSG00000115415 | 831 |
| ENSG00000080824 | ENSG00000144381 | 960 |
| ENSG00000080824 | ENSG00000198668 | 984 |
| ENSG00000080824 | ENSG00000254483 | 954 |
| ENSG00000080824 | ENSG00000086061 | 970 |
| ENSG00000080824 | ENSG00000181555 | 770 |
| ENSG00000080824 | ENSG00000228716 | 800 |
| ENSG00000128271 | ENSG00000148680 | 899 |
| ENSG00000128271 | ENSG00000175868 | 899 |
| ENSG00000149925 | ENSG00000198734 | 899 |
| ENSG00000149925 | ENSG00000278419 | 954 |
| ENSG00000149925 | ENSG00000111716 | 769 |
| ENSG00000170113 | ENSG00000133104 | 882 |
| ENSG00000148680 | ENSG00000175868 | 899 |
| ENSG00000072422 | ENSG00000079482 | 899 |
| ENSG00000072422 | ENSG00000057608 | 899 |
| ENSG00000153107 | ENSG00000197170 | 899 |
| ENSG00000153107 | ENSG00000115233 | 899 |
| ENSG00000248648 | ENSG00000258674 | 925 |
| ENSG00000145349 | ENSG00000162434 | 899 |
| ENSG00000145349 | ENSG00000198668 | 957 |
| ENSG00000145349 | ENSG00000115415 | 959 |
| ENSG00000145349 | ENSG00000118260 | 944 |
| ENSG00000144381 | ENSG00000228716 | 992 |
| ENSG00000144381 | ENSG00000077080 | 915 |
| ENSG00000144381 | ENSG00000270945 | 999 |
| ENSG00000110321 | ENSG00000125977 | 858 |
| ENSG00000179295 | ENSG00000119535 | 955 |
| ENSG00000179295 | ENSG00000162434 | 998 |
| ENSG00000179295 | ENSG00000163939 | 780 |
| ENSG00000179295 | ENSG00000134545 | 955 |
| ENSG00000179295 | ENSG00000115415 | 998 |
| ENSG00000179295 | ENSG00000171862 | 703 |
| ENSG00000179295 | ENSG00000117400 | 841 |
| ENSG00000179295 | ENSG00000030304 | 756 |
| ENSG00000179295 | ENSG00000264982 | 727 |
| ENSG00000238172 | ENSG00000237169 | 951 |
| ENSG00000238172 | ENSG00000251354 | 952 |
| ENSG00000009954 | ENSG00000077097 | 955 |
| ENSG00000119535 | ENSG00000162434 | 977 |
| ENSG00000119535 | ENSG00000115415 | 729 |
| ENSG00000162434 | ENSG00000249624 | 997 |
| ENSG00000162434 | ENSG00000268510 | 753 |
| ENSG00000162434 | ENSG00000115415 | 998 |
| ENSG00000162434 | ENSG00000117400 | 921 |
| ENSG00000162434 | ENSG00000134954 | 902 |
| ENSG00000162434 | ENSG00000152969 | 831 |
| ENSG00000249624 | ENSG00000115415 | 997 |
| ENSG00000236444 | ENSG00000171862 | 761 |
| ENSG00000236444 | ENSG00000254483 | 899 |
| ENSG00000175868 | ENSG00000075073 | 756 |
| ENSG00000175868 | ENSG00000175206 | 774 |
| ENSG00000175868 | ENSG00000241258 | 889 |
| ENSG00000271215 | ENSG00000237169 | 731 |
| ENSG00000269403 | ENSG00000196177 | 863 |
| ENSG00000175216 | ENSG00000196116 | 899 |
| ENSG00000175216 | ENSG00000113712 | 899 |
| ENSG00000175216 | ENSG00000077380 | 899 |
| ENSG00000175216 | ENSG00000227755 | 899 |
| ENSG00000054118 | ENSG00000130772 | 899 |
| ENSG00000268510 | ENSG00000115415 | 848 |
| ENSG00000197299 | ENSG00000067369 | 781 |
| ENSG00000133104 | ENSG00000078747 | 920 |
| ENSG00000260886 | ENSG00000108946 | 833 |
| ENSG00000260886 | ENSG00000151224 | 899 |
| ENSG00000260886 | ENSG00000117400 | 704 |
| ENSG00000260886 | ENSG00000118260 | 955 |
| ENSG00000197971 | ENSG00000198668 | 948 |
| ENSG00000197170 | ENSG00000120705 | 790 |
| ENSG00000197170 | ENSG00000115233 | 999 |
| ENSG00000146918 | ENSG00000138180 | 832 |
| ENSG00000283398 | ENSG00000067064 | 978 |
| ENSG00000163939 | ENSG00000077080 | 765 |
| ENSG00000198668 | ENSG00000251143 | 912 |
| ENSG00000198668 | ENSG00000122786 | 993 |
| ENSG00000198668 | ENSG00000198947 | 881 |
| ENSG00000198668 | ENSG00000169213 | 930 |
| ENSG00000198668 | ENSG00000124493 | 833 |
| ENSG00000198668 | ENSG00000090889 | 903 |
| ENSG00000198668 | ENSG00000030304 | 734 |
| ENSG00000198668 | ENSG00000102699 | 899 |
| ENSG00000198668 | ENSG00000072571 | 927 |
| ENSG00000198668 | ENSG00000173039 | 936 |
| ENSG00000198668 | ENSG00000118260 | 899 |
| ENSG00000198668 | ENSG00000185313 | 800 |
| ENSG00000198668 | ENSG00000117152 | 960 |
| ENSG00000285238 | ENSG00000196591 | 998 |
| ENSG00000197487 | ENSG00000135829 | 768 |
| ENSG00000285625 | ENSG00000198900 | 719 |
| ENSG00000285625 | ENSG00000065328 | 899 |
| ENSG00000285625 | ENSG00000283544 | 999 |
| ENSG00000263639 | ENSG00000101911 | 938 |
| ENSG00000108946 | ENSG00000256879 | 902 |
| ENSG00000108946 | ENSG00000138071 | 746 |
| ENSG00000256879 | ENSG00000143199 | 903 |
| ENSG00000251143 | ENSG00000090889 | 899 |
| ENSG00000251143 | ENSG00000102699 | 899 |
| ENSG00000113712 | ENSG00000077380 | 899 |
| ENSG00000256349 | ENSG00000187686 | 725 |
| ENSG00000120705 | ENSG00000237169 | 938 |
| ENSG00000120705 | ENSG00000125977 | 853 |
| ENSG00000120705 | ENSG00000256552 | 919 |
| ENSG00000115415 | ENSG00000030304 | 944 |
| ENSG00000115415 | ENSG00000134954 | 878 |
| ENSG00000115415 | ENSG00000173039 | 980 |
| ENSG00000115415 | ENSG00000118260 | 960 |
| ENSG00000115415 | ENSG00000144895 | 899 |
| ENSG00000198900 | ENSG00000077097 | 967 |
| ENSG00000237169 | ENSG00000251354 | 948 |
| ENSG00000237169 | ENSG00000256552 | 995 |
| ENSG00000198947 | ENSG00000070061 | 776 |
| ENSG00000198947 | ENSG00000284986 | 979 |
| ENSG00000174125 | ENSG00000254905 | 818 |
| ENSG00000065328 | ENSG00000283544 | 899 |
| ENSG00000150275 | ENSG00000169213 | 899 |
| ENSG00000136628 | ENSG00000133706 | 961 |
| ENSG00000143493 | ENSG00000070061 | 899 |
| ENSG00000074706 | ENSG00000268465 | 987 |
| ENSG00000135829 | ENSG00000102908 | 832 |
| ENSG00000118217 | ENSG00000118260 | 915 |
| ENSG00000160783 | ENSG00000173039 | 899 |
| ENSG00000196177 | ENSG00000255967 | 945 |
| ENSG00000187908 | ENSG00000171862 | 840 |
| ENSG00000178104 | ENSG00000204422 | 899 |
| ENSG00000155380 | ENSG00000088386 | 780 |
| ENSG00000155380 | ENSG00000111716 | 750 |
| ENSG00000065135 | ENSG00000172380 | 800 |
| ENSG00000166197 | ENSG00000118260 | 827 |
| ENSG00000162398 | ENSG00000250709 | 720 |
| ENSG00000138180 | ENSG00000090889 | 871 |
| ENSG00000138180 | ENSG00000072571 | 928 |
| ENSG00000171862 | ENSG00000107789 | 837 |
| ENSG00000171862 | ENSG00000171490 | 844 |
| ENSG00000171862 | ENSG00000173039 | 910 |
| ENSG00000171862 | ENSG00000181555 | 949 |
| ENSG00000084073 | ENSG00000086061 | 756 |
| ENSG00000124767 | ENSG00000139684 | 850 |
| ENSG00000251354 | ENSG00000138071 | 871 |
| ENSG00000251354 | ENSG00000256552 | 820 |
| ENSG00000124664 | ENSG00000206075 | 744 |
| ENSG00000124493 | ENSG00000117152 | 752 |
| ENSG00000090889 | ENSG00000102699 | 899 |
| ENSG00000090889 | ENSG00000072571 | 859 |
| ENSG00000188822 | ENSG00000135898 | 854 |
| ENSG00000070061 | ENSG00000083635 | 899 |
| ENSG00000125977 | ENSG00000256552 | 906 |
| ENSG00000125977 | ENSG00000144895 | 942 |
| ENSG00000158571 | ENSG00000278419 | 960 |
| ENSG00000232307 | ENSG00000117152 | 833 |
| ENSG00000088386 | ENSG00000118260 | 815 |
| ENSG00000124440 | ENSG00000181555 | 899 |
| ENSG00000254483 | ENSG00000227755 | 899 |
| ENSG00000205339 | ENSG00000227755 | 899 |
| ENSG00000257335 | ENSG00000101938 | 758 |
| ENSG00000134954 | ENSG00000118260 | 816 |
| ENSG00000254905 | ENSG00000251201 | 955 |
| ENSG00000050405 | ENSG00000254732 | 899 |
| ENSG00000196591 | ENSG00000173039 | 939 |
| ENSG00000196591 | ENSG00000118260 | 889 |
| ENSG00000196591 | ENSG00000077097 | 764 |
| ENSG00000101911 | ENSG00000165609 | 899 |
| ENSG00000173039 | ENSG00000118260 | 873 |
| ENSG00000181555 | ENSG00000118260 | 949 |
| ENSG00000181555 | ENSG00000111716 | 951 |
| ENSG00000118260 | ENSG00000111716 | 916 |
| ENSG00000118260 | ENSG00000102908 | 704 |
